# Microfluidics‐Based Microcarriers for Live‐Cell Delivery

**DOI:** 10.1002/advs.202414410

**Published:** 2025-04-04

**Authors:** Zhonglin Fang, Xinyuan Yang, Chong Wang, Luoran Shang

**Affiliations:** ^1^ Shanghai Xuhui Central Hospital Zhongshan‐Xuhui Hospital and the Shanghai Key Laboratory of Medical Epigenetics the International Co‐laboratory of Medical Epigenetics and Metabolism (Ministry of Science and Technology) Institutes of Biomedical Sciences Fudan University Shanghai 200032 China

**Keywords:** cell therapy, live‐cell delivery, microcarriers, microfluidics

## Abstract

Live‐cell therapy has emerged as a revolutionary treatment modality, providing a novel therapeutic avenue for intractable diseases. However, a major challenge in live‐cell therapy is to maintain live‐cell viability and efficacy during the treatment. Microcarriers are crucial for enhancing cell retention, viability, and functions by providing a protective scaffold and creating a supportive environment for live‐cell proliferation and metabolism. For microcarrier construction, the microfluidic technology demonstrates excellent characteristics in terms of controllability over microcarrier size and morphology as well as potential for high‐throughput production. To date, multiple live‐cell delivery microcarrier types (e.g., microspheres, microfibers, and microneedles) are prepared via microfluidic liquid templates to meet different therapeutic needs. In this review, recent developments in microfluidics‐based microcarriers for live‐cell delivery are presented. It is focused on categorizing the structural design of microfluidic‐derived cell‐laden microcarriers, and summarizing various therapeutic applications. Finally, an outlook is provided on the future challenges and opportunities in this field.

## Graphical abstract

In this review, recent advancements in microfluidics‐based microcarriers for live‐cell delivery are highlighted. The structural designs of microfluidic‐derived cell‐laden microcarriers are systematically categorized, emphasizing innovative approaches in the fabrication of microspheres, microfibers, and microneedles. A comprehensive discussion of their therapeutic applications is also provided. Finally, an outlook is offered on the future challenges and opportunities in this field.

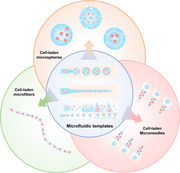

## 1 Introduction

Live‐cell therapy involves live‐cell administration against diseases as therapeutic agents.^[^ ^1^ ^]^ By using live cells to repair, replace, or enhance diseased cells, this approach offers a new treatment alternative for conditions such as cancer,^[^ ^2^, ^3^, ^4^ ^]^ tissue damage,^[^ ^5^, ^6^, ^7^, ^8^ ^]^ intestinal disease,^[^ ^9^, ^10^ ^]^ metabolic disorders,^[^ ^11^, ^12^ ^]^ etc. The therapeutic cells either replace impaired cells directly or secrete bioactive molecules in response to microenvironmental changes.^[^ ^13^, ^14^ ^]^ The unique characteristics of live‐cell therapy involve a more personalized treatment strategy, contrasting sharply with traditional pharmaceutical modalities.^[^ ^15^ ^]^ Live‐cell therapy could be tailored to meet the specific needs of each patient, enhancing the natural self‐repair capabilities of the body, and providing a more dynamic treatment option.^[^ ^16^ ^]^ While traditional drugs often exert their effects by directly inhibiting or activating specific biological pathways, potentially limiting their efficacy against complex diseases, live‐cell therapy harnesses intercellular signaling to activate various biological mechanisms within the body, resulting in a more comprehensive therapeutic effect.^[^ ^17^, ^18^ ^]^ For instance, stem cells hold significant promise in treating tissue damage and injury based on their regenerative potential, as they can repair or replace damaged tissues by differentiating into specific cell types (e.g., muscle, nerve, or cartilage cells).^[^ ^19^, ^20^ ^]^ This versatility presents a paradigm shift in the approach to chronic and degenerative diseases, emphasizing the intrinsic healing capacity of the body. Moreover, live‐cell therapeutics represented by chimeric antigen receptor (CAR) T cells have revolutionized tumor therapy. With six CAR‐T therapeutics approved by the Food and Drug Administration to date, this approach demonstrates the way genetically modified T cells could yield significant clinical benefits in cancer treatment.^[^ ^21^, ^22^ ^]^

Although cell therapy is promising, effective live‐cell administrations is challenging considering the mechanical damage and immune attack the cells are exposed to, potentially resulting in reduced cell viability in the target tissues.^[^ ^23^, ^24^ ^]^ Of note, only 1–20% of the cells survive upon direct injection,^[^ ^25^ ^]^ and the transplanted cells tend to remain at the injection site only for a short time, resulting in limited therapeutic performance. To address these problems, various microcarriers have been developed, aiming at avoiding direct contact between the cells and the hostile environment, thereby minimizing cell damage during the delivery process.^[^ ^23^, ^26^, ^27^, ^28^ ^]^ In addition, microcarriers could provide an appropriate microenvironment for cell adhesion and proliferation, eventually improving live‐cell viability and therapeutic efficacy and reducing side effects.^[^ ^29^ ^]^ All these merits necessitate a facile design of the microcarrier structure, microcarrier is thus crucial for cell delivery. Traditional microcarrier preparation techniques include bulk emulsification, phase separation, spray drying, etc.^[^ ^30^, ^31^, ^32^ ^]^ However, these methods retain several limitations, such as reliance on batch processing, complex equipment, low throughput, and microparticle size polydispersity.^[^ ^28^, ^33^ ^]^ Furthermore, uneven mechanical stirring during the manufacturing process, along with the use of certain cytotoxic substance, might render these methods unsuitable for the in situ encapsulation of delicate live cells.

Microfluidics, a technology allowing for precise fluid manipulation within microchannels, is exceptional for continuous microcarrier production with controlled size and morphology.^[^ ^34^ ^]^ Due to their flexible fluid dynamics control, microcarriers could be generated by solidifying liquid templates including jets, droplets, complex emulsions, etc.^[^ ^32^ ^]^ With that, various microcarrier types have been constructed, including microspheres,^[^ ^35^, ^36^, ^37^ ^]^ microfibers,^[^ ^38^, ^39^ ^]^ and microneedles,^[^ ^40^ ^]^ each retaining its own advantages. Live cells could be easily loaded by simply mixing into the liquid phases prior to solidification or co‐culturing with the produced microcarriers.^[^ ^41^ ^]^ Microcarriers prepared via the microfluidic method could yield a very narrow particle size distribution, thereby ensuring consistency in various properties and behaviors, such as live‐cell protective effects, degradation rates, and bioactive substance release rates.^[^ ^42^ ^]^ Moreover, the physicochemical properties of the microcarriers (including porosity, mechanical property, surface moieties, and biological responses) could be finely tuned through material component selection or combining with other techniques, thereby enriching their functionality.

**Figure 1:**
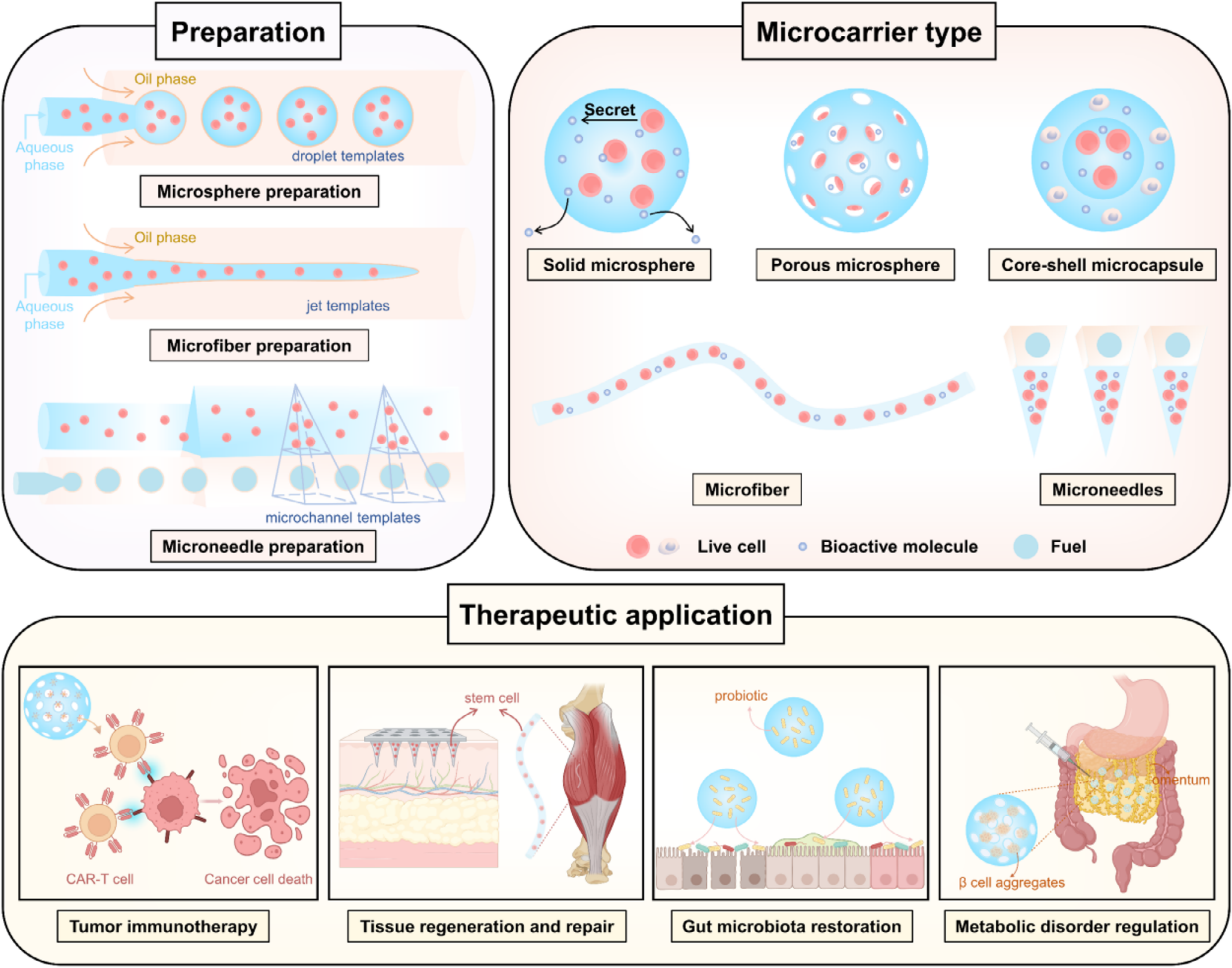
Schematic illustration of microfluidics‐based cell delivery microcarriers for disease treatment. Graphic created with BioRender.com.

Here, we provide a concise overview of microfluidics‐based cell delivery microcarriers and their diverse therapeutic applications (**Figure** **1**). We begin with typical cell‐laden microcarrier structures, focusing on their design, fabrication, and performance, while also incorporating fundamental microfluidics concepts. Next, we systematically discuss the recent progress of cell‐laden microcarriers in disease treatment. Finally, we highlight the remaining challenges of microfluidics‐based live‐cell delivery technology and present future trends in this field.

## 2 Structural Design of Microfluidics‐Based Live‐Cell Microcarriers

In microfluidic systems, the geometric characteristics of microcarriers (e.g., shape and size) are primarily governed by three key determinants: 1) microchannel design parameters, 2) fluid properties and compositional ingredients, and 3) operational conditions including flow rates and external field applications.^[^ ^43^, ^44^, ^45^ ^]^ Microcarriers with diverse structures can meet specific demands in cell delivery. What's more, the great flexibility in the precursor selection and cross‐linking protocols enables the integration of multifunctional properties into these microcarriers. In this section, we categorize the structural design and fabrication methods of microfluidics‐based live‐cell microcarriers.

### 2.1 Microspheres and Microcapsules

A hallmark feature of microfluidic technology is its ability to continuously produce monodisperse droplets. When two immiscible fluids flow through a microfluidic channel junction, droplet formation occurs through the interplay of shear forces and the interface tension.^[^ ^46^ ^]^ Besides, adding surfactants to either the continuous or dispersed phase can lower the energy needed for droplet formation and enhance droplet stability.^[^ ^47^ ^]^ The generated droplets are excellent precursors, which can be cross‐linked into microspheres or microcapsules by chemical gelation, thermal gelation, or ionic gelation.^[^ ^48^, ^49^ ^]^

#### 2.1.1 Solid Microspheres

Three representative microchannel geometries are commonly used for droplet generation in microfluidics: T‐junction, coaxial flow, and flow‐focusing. Droplets can form passively without external agitation or actively under electric, magnetic, centrifugal, or other forces.^[^ ^50^, ^51^, ^52^ ^]^ Notably, the methods employed for droplet generation serve as the foundation for fabricating various cell‐laden microspheres. Among numerous microsphere types, solid microspheres feature the simplest structure and easiest fabrication process. Here, we exemplify with the most basic solid microspheres and delve into recent progress in employing these methods for the generation of cell delivery microcarriers.

**Figure 2:**
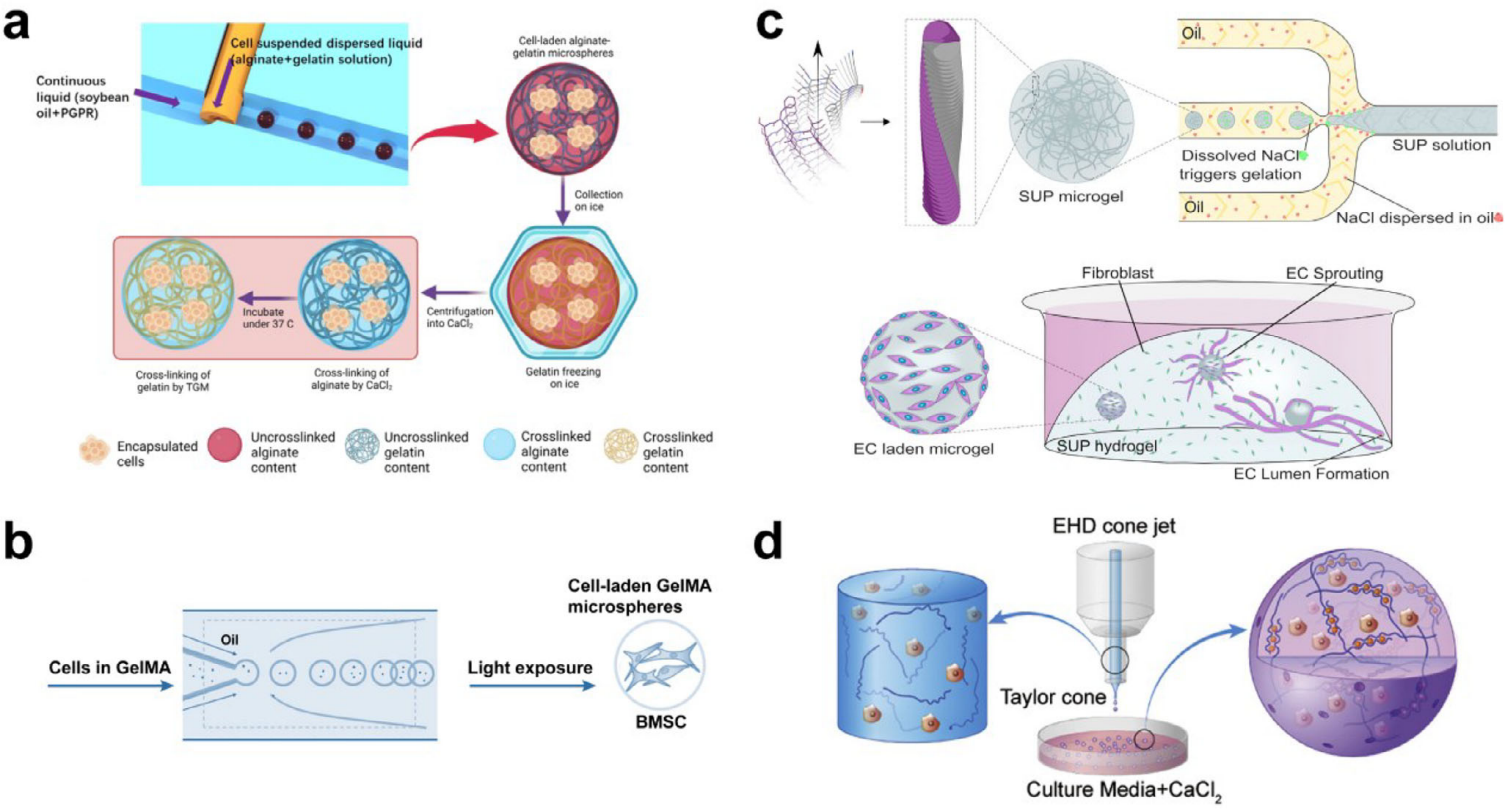
Representative microfluidic setups for generating cell‐laden solid microspheres. a) A modified capillary‐embedded “step‐T‐junction” device for high‐throughput generation of monodisperse cell‐laden alginate‐gelatin solid microspheres. Reproduced with permission.^[^ ^54^ ^]^ Copyright 2022, MDPI. b) Coaxial flow microchannels for the formation of BMSC‐laden GelMA solid microspheres. Reproduced with permission.^[^ ^36^ ^]^ Copyright 2016, John Wiley and Sons. c) A flow‐focusing microfluidic platform for the fabrication of SUP solid microspheres loading HUVECs. Reproduced with permission.^[^ ^37^ ^]^ Copyright 2021, American Chemical Society. d) Microfluidic electrospray technology for the fabrication of alginate solid microspheres loaded with VEGF‐expressing HEK293T cells. Reproduced with permission.^[^ ^57^ ^]^ Copyright 2020, Elsevier.

In traditional T‐junction microchannels, droplets generated in squeezing or dripping modes typically exhibit lower throughput, while droplets generated in the jetting mode with higher throughput showed suboptimal monodispersity.^[^ ^53^ ^]^ The dilemma is particularly acute when enhancing viscosity of the dispersed phase. To overcome this challenge, Ling et al. developed cell‐laden solid microspheres by employing a novel “step‐T‐junction” microchannel with a perpendicularly embedded microcapillary (**Figure** **2**a).^[^ ^54^ ^]^ This step‐T‐junction channel provides dual perturbations onto the dispersed phase, facilitating the shearing of high‐viscosity, cell‐laden alginate/gelatin solutions and allowing for high‐throughput generation of microspheres with satisfactory monodispersity. Such a design enables the production of microspheres with tightly controlled size distributions, which is critical for ensuring consistent mass transfer rates and mechanical properties in downstream applications such as 3D cell culture scaffolds and live‐cell delivery systems.

Compared to the T‐junction geometry, coaxial flow and flow‐focusing geometries are supposed to be more suitable for encapsulating fragile objects like cells.^[^ ^55^ ^]^ As a typical example, Zhao et al. employed biocompatible methacrylated gelatin (GelMA) as the material component, and generated uniform microspheres encapsulating bone marrow‐derived mesenchymal stem cells (BMSCs) through a coaxial flow microfluidic device (Figure 2b).^[^ ^36^ ^]^ By modifying the flow rate ratio (GelMA solution / oil phase) to 0.1, they achieved solid microspheres of ≈160 µm, an optimal size for accommodating a substantial number of cells and, at the same time, facilitating efficient oxygen and nutrient exchange. Additionally, an optimized UV cross‐linking time of 20 s was employed to ensure adequate GelMA cross‐linking while preserving cell viability. In addition to encapsulating cells within microspheres, it's also possible to cultivate cells on the surface of microspheres. As depicted in Figure 2c, Hauser and colleagues used a flow‐focusing microfluidic chip to create self‐assembling ultrashort peptides (SUP) hydrogel microspheres as microcarriers for diverse cell types.^[^ ^37^ ^]^ The peptides are amphiphilic and can self‐assemble into extracellular matrix (ECM)‐like hydrogels. The gelation of SUP solution droplets was triggered by simply introducing sodium chloride into the continuous oil phase. Cells were let grow at the microspheres’ surface in order to achieve faster proliferation than being encapsulated.

In contrast to the above platforms that rely on immiscible aqueous and oil phase fluids to generate droplets, the microfluidic electrospray approach does not involve the use of oil as the continuous phase. Instead, it relies on an electric field, provided by a high‐pressure power source to induce the formation of a “Taylor cone” at the tip of the fluid, which subsequently transitions into falling droplets.^[^ ^56^ ^]^ The oil‐free system offers a mild environment for live‐cell loading. Compared with photoinitiated polymerization, microfluidic electrospray has been more commonly adopted for generating droplets that can be rapidly cross‐linked with ions in a collection bath. Shen et al. employed this technique to generate sodium alginate microspheres loaded with engineered HEK293T cells that over‐produce vascular endothelial growth factor (VEGF) (Figure 2d).^[^ ^57^ ^]^ The cell density at 10^7^ cells mL^−1^ showed the best proliferation. It was shown that a 1% concentration of alginate resulted in the lowest cell death when applied to in vivo therapy. Additionally, dead cells were predominantly located at the edges of the solid microspheres, and the cells within were not significantly affected by mechanical damage or environmental stress.

#### 2.1.2 Porous Microspheres

Porous microspheres are defined by the presence of interconnected pores within their structure, distinguishing them from conventional solid microspheres through their substantially enhanced specific surface area. This pore network provides permeability for oxygen and nutrient transport into the microsphere interior.^[^ ^58^ ^]^ Cells can also more easily access essential nutrients, release metabolic waste products, and interact with the surrounding environment.^[^ ^59^ ^]^ The size of the pores should be controlled within an appropriate range.^[^ ^60^ ^]^ For example, excessively large pores may reduce effective surface area for cell attachment and growth, as well as hinder the maintenance of the cellular microenvironment.

**Figure 3:**
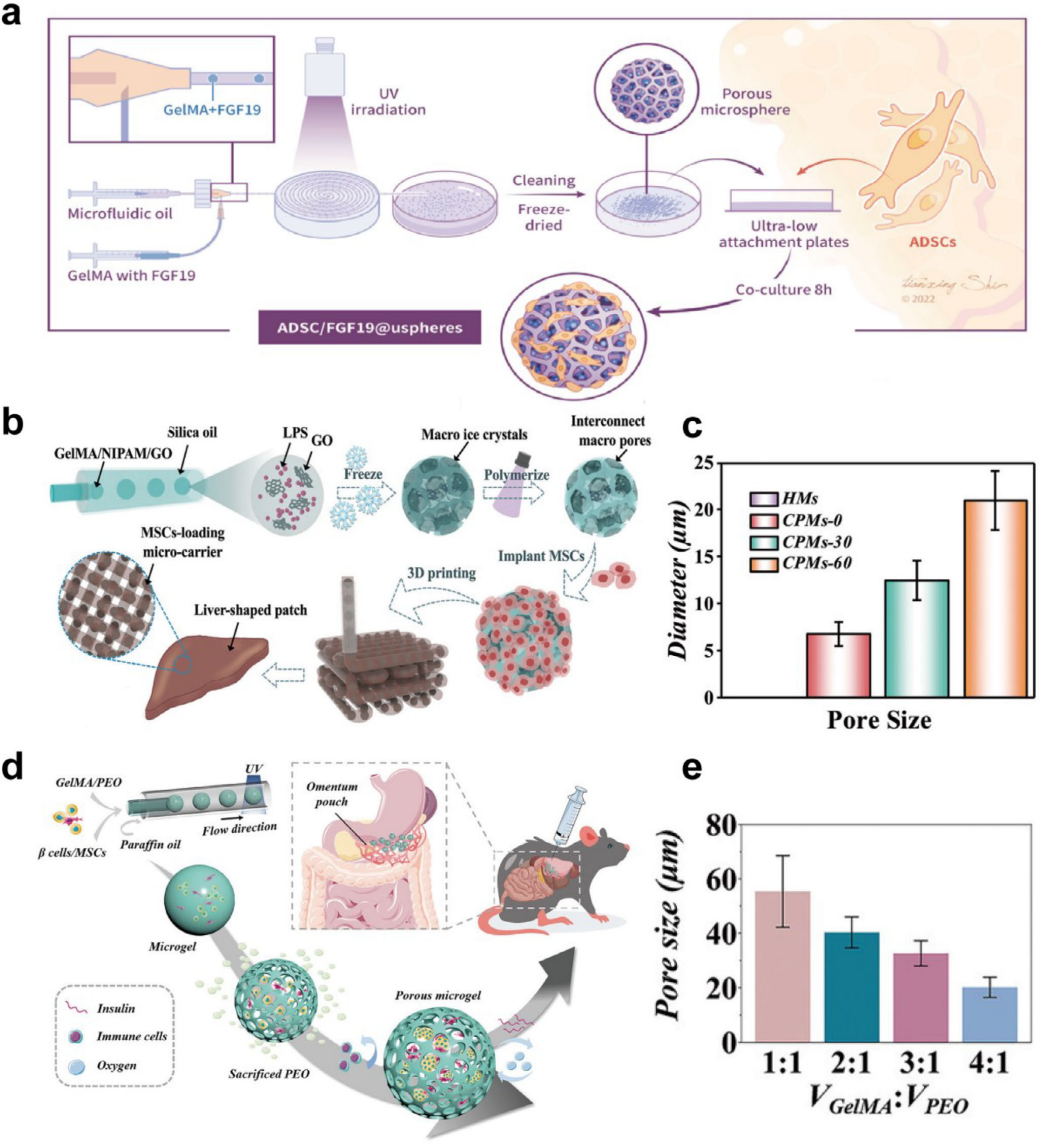
Different approaches for preparing cell‐laden porous microspheres based on microfluidics. a) GelMA microspheres with high porosity via freeze‐drying as microcarriers for ADSCs. Reproduced with permission.^[^ ^61^ ^]^ Copyright 2023, The Authors, published by Elsevier. b) GelMA porous microspheres loaded with MSCs were fabricated through gradient freezing, and then were 3D‐printed into liver‐shaped patches. c) Adjusting the first‐step freezing time to obtain CPMs with different pore diameter. Reproduced with permission.^[^ ^63^ ^]^ Copyright 2022, John Wiley and Sons. d) Utilizing PEO as a sacrificial template for creating pores in cell‐loaded GelMA microspheres. e) Different pore sizes formed with varying GelMA to PEO volume ratios from 1:1 to 4:1. Reproduced with permission.^[^ ^64^ ^]^ Copyright 2023, John Wiley and Sons.

Freeze‐drying, gradient freezing and the use of sacrificial template are common techniques for porous microsphere fabrication. During the freeze‐drying process, moisture directly sublimates from the interior of the microspheres under reduced pressure and low‐temperature conditions, leaving voids and thus creating porous structures. As an example, Si and colleagues constructed GelMA microspheres encapsulating fibroblast growth factor 19 (FGF19) (**Figure** **3**a).^[^ ^61^ ^]^ After freeze‐drying, porous microspheres with pore diameters ranging from 20–40 µm were obtained. This structure provided large surface area for the loading and adhesion of adipose‐derived stem cells (ADSCs). In comparison with culture plates, ADSCs exhibited higher‐density proliferation on porous GelMA microspheres. Differently, the gradient‐freezing method has been adopted to regulate the pore size.^[^ ^62^ ^]^ Following a similar procedure, Wang et al. created cryogel porous microcarrier (CPM) (Figure 3b).^[^ ^63^ ^]^ First, the GelMA composite pregel droplets generated by microfluidics were frozen at ‐20°C, leading to separation of the frozen ice crystals and the surrounding unfrozen pregel. Then, liquid nitrogen (‐196°C) was poured into the system to stop ice crystal expansion, and UV light was applied to solidify the gel, resulted in the formation of microspheres with interconnected macropores. By extending the first‐step freezing time, progressively larger ice crystal sizes can be achieved, as shown in Figure 3c.

The sacrificial template method typically includes the addition of a porogen to the pregel. Once the microspheres are created, the porogen can be removed to form pores. Based on this, Sun et al. mixed poly (ethylene oxide) (PEO) into a GelMA pregel solution containing islet β cells and MSCs (Figure 3d).^[^ ^64^ ^]^ After microspheres were generated from microfluidic channels, PEO was removed by immersion in PBS solution for 24 h. The pore size was directly correlated with the PEO proportion in the system (Figure 3e). In addition to PEO, gelatin, ammonium bicarbonate, SiO_2_ crystalline template, and gas molecules can serve as porogens for constructing porous microspheres.^[^ ^60^, ^65^, ^66^, ^67^, ^68^ ^]^

#### 2.1.3 Core‐Shell Microcapsules

Core‐shell microcapsules comprise a core and a surrounding shell layer. Typically, these microcarriers are created using double emulsion droplets as templates.^[^ ^35^ ^]^ Microfluidics enables the precise creation of core‐shell microcarriers with uniform size and adjustable shell thickness.^[^ ^69^ ^]^ Theoretically, cells can be encapsulated within the core or the shell layer according to specific requirements. Alternatively, two different types of cells can be separately enclosed within the core and shell layer.^[^ ^70^ ^]^

**Figure 4:**
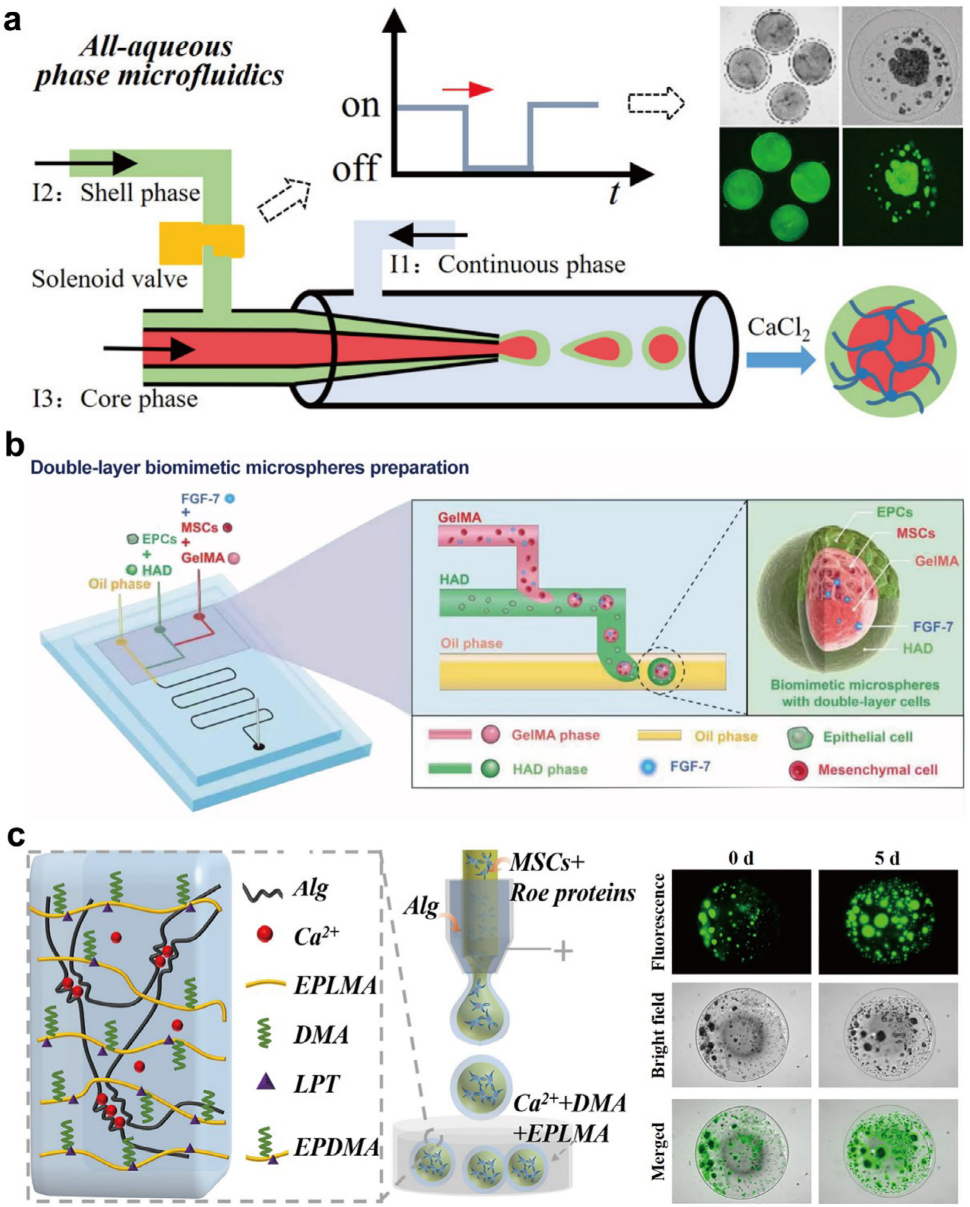
Microfluidic construction of cell‐laden core‐shell microcapsules. a) All‐aqueous‐phase microfluidics for constructing cell‐laden core‐shell microcapsules. Reproduced with permission.^[^ ^73^ ^]^ Copyright 2019, American Chemical Society. b) Microfluidic preparation of microcapsules encapsulating murine MSCs and murine EPCs in the core and shell, respectively. Reproduced with permission.^[^ ^74^ ^]^ Copyright 2023, IOP Publishing. c) MSCs‐laden core‐shell microcapsules generated by microfluidic electrospray. Reproduced with permission.^[^ ^75^ ^]^ Copyright 2023, National Academy of Sciences.

For core‐shell microcarriers utilized for live‐cell delivery, it is recommended that both the core and shell provide a mild environment to ensure cell viability. Unlike traditional emulsions that typically involve oil and water phases, the all‐aqueous‐phase system can be a good choice. For some specific aqueous components, when their concentration exceeds a critical threshold, the system's interaction energy can surpass the Gibbs free energy for mixing, thus effectively preventing them from blending.^[^ ^71^, ^72^ ^]^ Following this principle, Zhu et al. utilized a high concentration of polyethylene glycol, an alginate‐dextran mixture, and ADSC‐suspended carboxymethyl cellulose (CMC) sodium as the fluid for the continuous, shell, and core phase, respectively (**Figure** **4**a).^[^ ^73^ ^]^ They introduced an oscillating valve to agitate the shell phase fluid so as to assist the emulsification process. This helped to achieve one‐step synthesis of all‐aqueous cellular core‐shell microcapsules. This oil‐free production method eliminates the need for complex washing steps and allows for the direct cultivation of ADSCs. The encapsulated cells can proliferate into cell aggregates within the microcapsules for a continuous week. In a separate study by Hu and co‐workers, microcapsules with a core of murine MSCs‐encapsulated GelMA and a shell of murine epidermal cells (EPCs)‐catechol grafted hyaluronic acid were fabricated via water‐in‐water‐in‐oil emulsions (Figure 4b).^[^ ^74^ ^]^ After encapsulation with the core‐shell microcarriers, the cells retained their characteristic shapes, with their morphology well‐preserved. Following 9 days of in vitro culture, the core‐shell microcapsules still retained over 80% cell viability, whereas the separately cultured cell spheroids began to show central necrosis after 5 days.

Microfluidic electrospray provides an alternative way to create cell‐laden core‐shell microcapsules. Chen et al. presented core‐shell microcapsules for MSCs encapsulation, as depicted in Figure 4c.^[^ ^75^ ^]^ Because of the hydrodynamic focusing effect, the inner phase was enveloped by the outer phase during mixing. Microcarriers with a liquid core and a dual‐network shell were formed in a collection pool. The roe protein provided a continuous supply of nutrients to the cells, and the embryo‐like structure of the microcapsules offered a biocompatible environment.

### 2.2 Microfibers

Different from microspheres that are formed from emulsion droplet templates, microfluidic‐derived microfibers are typically formed from jet templates.^[^ ^76^, ^77^ ^]^ The microfluidic method not only enhances the uniformity of the fiber diameters but also enables the incorporation of functional materials during the formation process. As a result, microfluidic‐derived microfibers can exhibit unique characteristics, such as enhanced mechanical strength, improved mass transfer rates, and the ability to support cellular adhesion, making them particularly valuable for applications in live‐cell delivery systems.^[^ ^78^, ^79^, ^80^ ^]^ In addition, microfibers can be made flexible, and even be folded, bundled, curled, and woven into intricate fibrous architectures to mimic tissues like blood vessels, nerves, and muscles in the human body.^[^ ^81^, ^82^ ^]^ Therefore, cell‐laden microfibers are highly favorable for tissue regeneration and repair.

**Figure 5:**
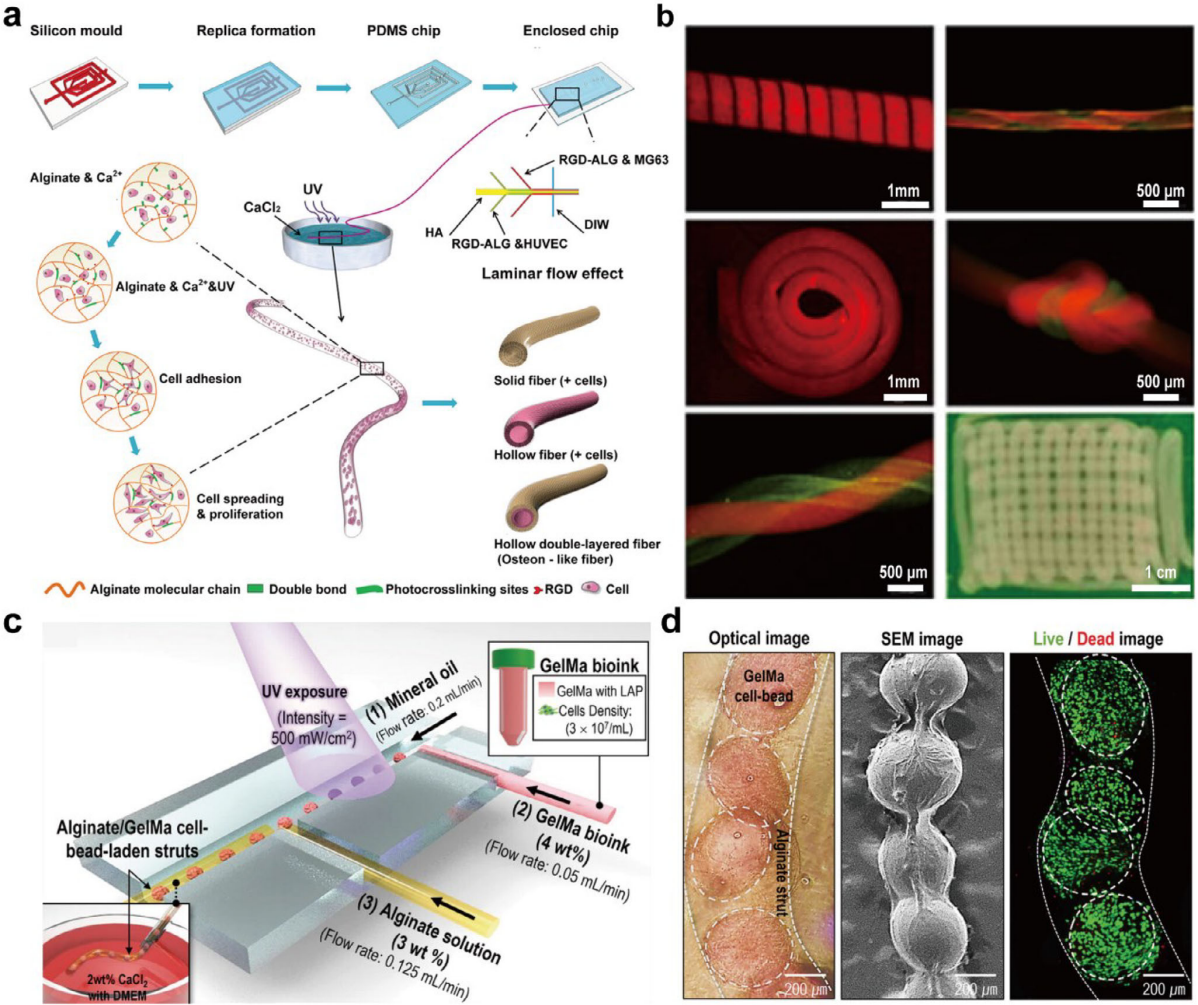
Microfluidics‐based strategies for cell‐laden microfibers production. a) Schematic illustration of the construction of the PDMS microfluidic chip and its application in creating diverse‐shaped microfibers with RGD‐modified alginate. b) Microscopic images of diverse larger‐scale fiber assemblies. Reproduced with permission.^[^ ^83^ ^]^ Copyright 2017, American Chemical Society. c) Microfluidic device employed for developing alginate/GelMA cell bead‐laden microfibers. d) Characterizations of the cell bead‐laden microfibers. Reproduced with permission.^[^ ^84^ ^]^ Copyright 2021, John Wiley and Sons.

Microfibers exhibit long and slender structures and are able to accommodate a sufficient number of cells to fulfill functions such as in vivo transplantation and tissue modeling. The hydrogel materials that make up the microfibers cannot naturally and fully replicate the cellular environment. Therefore, the key is to create a microenvironment in the microfibers that fosters cell‐to‐cell connections, enabling the cells to demonstrate their inherent morphology and functionality during 3D culture. One pioneering work was done by Onoe et al., who employed a dual‐coaxial microfluidic system to produce meter‐long tubular microfibers with core‐shell structure, where the shell comprised alginate and the core housed cells and ECM proteins.^[^ ^38^ ^]^ Despite a relatively long gelation time of the ECM proteins, their higher molecular weight as compared to the Ca‐alginate hydrogel's molecular weight cutoff ensured that they can remain confined within the shell, allowing sufficient time for proper gelation. During cell fiber formation, cells contacted each other, causing the initial ECM proteins to gradually disappear and be replaced by newly produced cell‐derived ECM. Finally, the alginate shell was degraded using alginate lyase to produce intact cell fibers. In another attempt, Wei et al. used a lithography‐based polydimethylsiloxane (PDMS) microfluidic chip for continuous fabrication of multilayered cell‐laden fibers (**Figure** **5**a).^[^ ^83^ ^]^ Methacrylated alginate grafted with the Arg‐Gly‐Asp (RGD) peptide was prepared, and fibers were formed by calcium ions cross‐linking. UV cross‐linking was also employed to further enhance the mechanical strength of the hydrogel. The RGD peptide promotes cell adhesion and biological activity. After the microfibers with sufficient strength were obtained, they could be assembled into larger‐scale by winding, twisting, or 3D printing, demonstrating their potential for forming complex tissues (Figure 5b).

In addition to this, the combination of microfluidic spinning and emulsification enables fabrication of composite fibers with microspheres embedded. Kim et al. presented a microfluidic chip with two T‐junctions to construct composite Ca‐alginate hydrogel microfibers with cell‐laden GelMA beads. Under UV light exposure, GelMA bioink with cells was dispersed into microbeads in a surrounding mineral oil. After flowing an alginate solution, the GelMA cell beads penetrated into this aqueous fluid, and the composite fibers were formed after cross‐linking in a bath containing calcium ions (Figure 5c).^[^ ^84^ ^]^ The results showed that cells were well‐survived within the microfibers (Figure 5d). It was confirmed that the cell‐laden beads could increase cell interactions to a level similar to cellular spheroids.

### 2.3 Microneedles

Microneedles are transdermal drug delivery devices composed of multiple micrometer‐sized fine needle tips. The height of the microneedles typically ranges from 100 to 2000 µm, allowing them to penetrate the stratum corneum and create micron‐sized channels for the precise delivery of bioactive compounds like chemical drugs, nanoparticles, nucleic acids, proteins, vaccines, and live cells to the dermal layer of the skin.^[^ ^27^, ^85^, ^86^, ^87^ ^]^ Compared to traditional needles, microneedles are less invasive and cause low trauma and pain, which significantly improves the compliance of patients.

**Figure 6:**
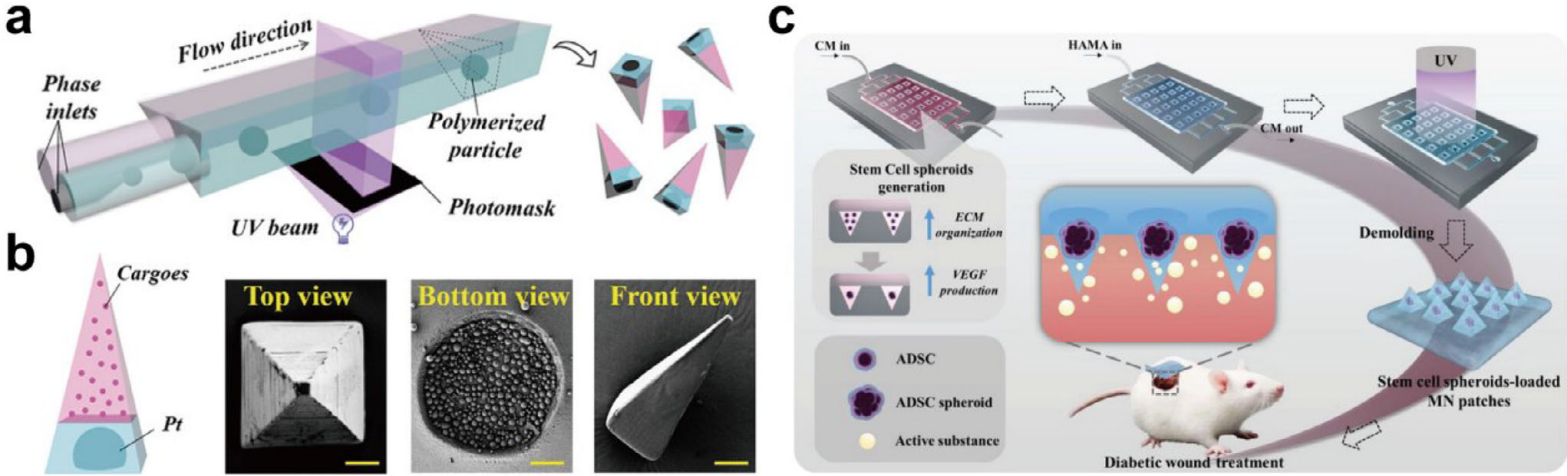
Constructing cell‐laden microneedle arrays with the aid of microfluidics. a) Schematic showing the design of the microfluidic device and the generation of the microneedles. b) Schematic illustration and SEM images of the Pt nanoparticles‐laden microneedles. Scale bars are 200, 50, and 100 µm in top view, bottom view, and front view, respectively. Reproduced with permission.^[^ ^40^ ^]^ Copyright 2023, John Wiley and Sons. c) Microfluidic template‐assisted fabrication of microneedle arrays loaded with ADSCs spheroids. Reproduced with permission.^[^ ^89^ ^]^ Copyright 2023, John Wiley and Sons.

Through the rational design of microfluidic chips and channels, microneedles can be directly fabricated using fluid templates.^[^ ^40^, ^88^, ^89^, ^90^, ^91^ ^]^ Cai et al. constructed a unique microfluidic device with three capillary tubes and one PDMS channel with a triangular cross section (**Figure** **6**a).^[^ ^40^ ^]^ GelMA solution was introduced into both the parallel phase and the outer phase. Ethylene glycol dimethacrylate solution dispersed with Pt nanoparticles was introduced into the inner phase. The polymerization process was initiated by directing UV light through a meticulously designed triangular photomask, a critical step that enabled the spatially controlled solidification of the polymer within the channels (Figure 6b). This strategy underscores the significance of light‐based techniques in microfabrication and showcases the potential for creating complex 3D microneedles with tailored geometries. For the loading of cells, Zhao and colleagues developed an innovative microneedle patch by a microfluidic template for in situ encapsulation of stem cell spheroids (Figure 6c).^[^ ^89^ ^]^ To prevent cell adhesion and promote the formation of spheroids, the chip surface was pre‐treated with hydrophobic f127. In this meticulous procedure, ADSC suspensions were injected into the microfluidic template, where cells were uniformly distributed in each microwell and aggregated at the bottom of the micropores to form a spheroidal cluster. The precise cellular arrangement improved the efficiency of subsequent operations and established a solid foundation for the consistency of cell quantity, state, and viability within each microneedle unit in the microchannel. Subsequently, methacrylated hyaluronic acid (HAMA) pregel was injected into the microfluidic template, followed by UV polymerization. After demolding, a microneedle array encapsulating ADSC spheroids was formed. During culture, the viability of ADSC spheroids in the microneedle array gradually increased and stabilized at about 92% after 72 h. The spheroids also displayed a well‐organized structure and distribution of actin filaments.

## 3 Therapeutic Applications of Microfluidics‐Based Cell Delivery Microcarriers

Microfluidics provides an excellent platform to create diverse cell‐laden microcarriers (**Table** **1**). Microcarriers offer a confined space for live‐cell protection. Some activation signals such as antibodies and cytokines can be affixed onto microcarriers to promote the proliferation of loaded cells or regulate cell fate.^[^ ^92^ ^]^ Cell‐laden microcarriers can also be chemically modified, surface‐coated, or co‐encapsulated with other active molecules to provide complementary functions.^[^ ^93^, ^94^ ^]^ Some microcarriers with sustained‐release effects enable bioactive molecules secreted by live cells to be released in a smooth manner.^[^ ^95^ ^]^ In this section, several typical applications of microfluidics‐based microcarriers are introduced, including the treatment of diseases such as tumors, tissue damage, intestinal diseases, and metabolic disorders.

**Table 1:** Representative examples of microfluidic‐derived cell‐laden microcarriers for disease treatment.

Structural types	Materials	Loaded cells	Disease	Key advantages	Refs.
Solid microspheres	GelMA	BMSCs	Bone defects	Biocompatibility, high cell viability	[36]
	Poly‐γ‐glutamic acid	*Lactobacillus acidophilus*	Inflam	Gastric acid‐resisting, inflammatory targeting	[96]
	Methacrylated dextran/Methacrylated tannic acid	*Escherichia coli* Nissle 1917	IBD	Colon‐targeting, intestinal mucosal adhesion	[97]
Porous microspheres	GelMA	ADSCs	Limb damage	Sustained release, enhanced delivery efficiency	[61]
	GelMA/*N*‐isopropylacrylamide	MSCs	Acute liver failure	Photothermal effect, thermoresponsive behavior, localized delivery	[63]
	PLGA	C2C12 cells	Skeletal muscle injury	Minimally invasive	[60]
	GelMA	Nucleus pulposus cells	Intervertebral disc degeneration	Inhibit overactive local inflammatory response	[93]
	PLGA	CAR‐T cells	Cervical tumor	Effectively stimulate the CAR‐T cells expansion, prolonging therapeutic effect	[98]
	Alginate	NK92‐MI cells	Skin cancer	Enable direct killing, sustained release of killing factors	[99]
	Poly(ethylene glycol) diacrylate/GelMA	β cells	Diabetes	Facilitate cell aggregation, enhanced exchange efficiency	[100]
Core‐shell microcapsules	GelMA/hyaluronic acid	MSCs/EPCs	Hair loss	Aqueous phase of the core and shell layer, superior tissue adhesion properties	[74]
	Alginate/ epsilon‐poly‐L‐lysine‐graft‐methacrylamide/dopamine methacrylamide	MSCs	IBD	Wet adhesive, immune isolation, enhanced mechanical strength and stability	[75]
	Alginate/cellulose nanocrystals	BMSCs	Bone defects	Allow for substance exchange, avoid mechanical damage	[101]
	Poly(ethylene glycol) diacrylate	UCB‐MSCs	IBD	Isolate from gastric acid, with responsive release in the intestine	[102]
	Alginate/CMC	*Lactobacillus*/ *Bacillus subtilis*	Metabolic syndrome	Two types of cells have a mutually beneficial symbiosis	[103]
Microfibers	Alginate/agarose	Primary islet cell	Diabetes	Tissue simulation, high level assembly capability, removability	[38]
	Alginate/GelMA	hASCs	Volumetric Muscle Loss	Tissue simulation, excellent mechanical properties, long‐term retention in the body	[84]
	Alginate/collagen	RUVECs	Calvarial defects	mimicking blood vessels, excellent perfusion and penetration properties	[104]
	Alginate/polyacrylamide	β cells	Diabetes	Improved mechanical strength	[105]
Microneedles	HAMA	ADSCs	Diabetic wound	Uniform cell distribution and accurate delivery of bioactive substances	[89]

### 3.1 Tumor Immunotherapy

**Figure 7:**
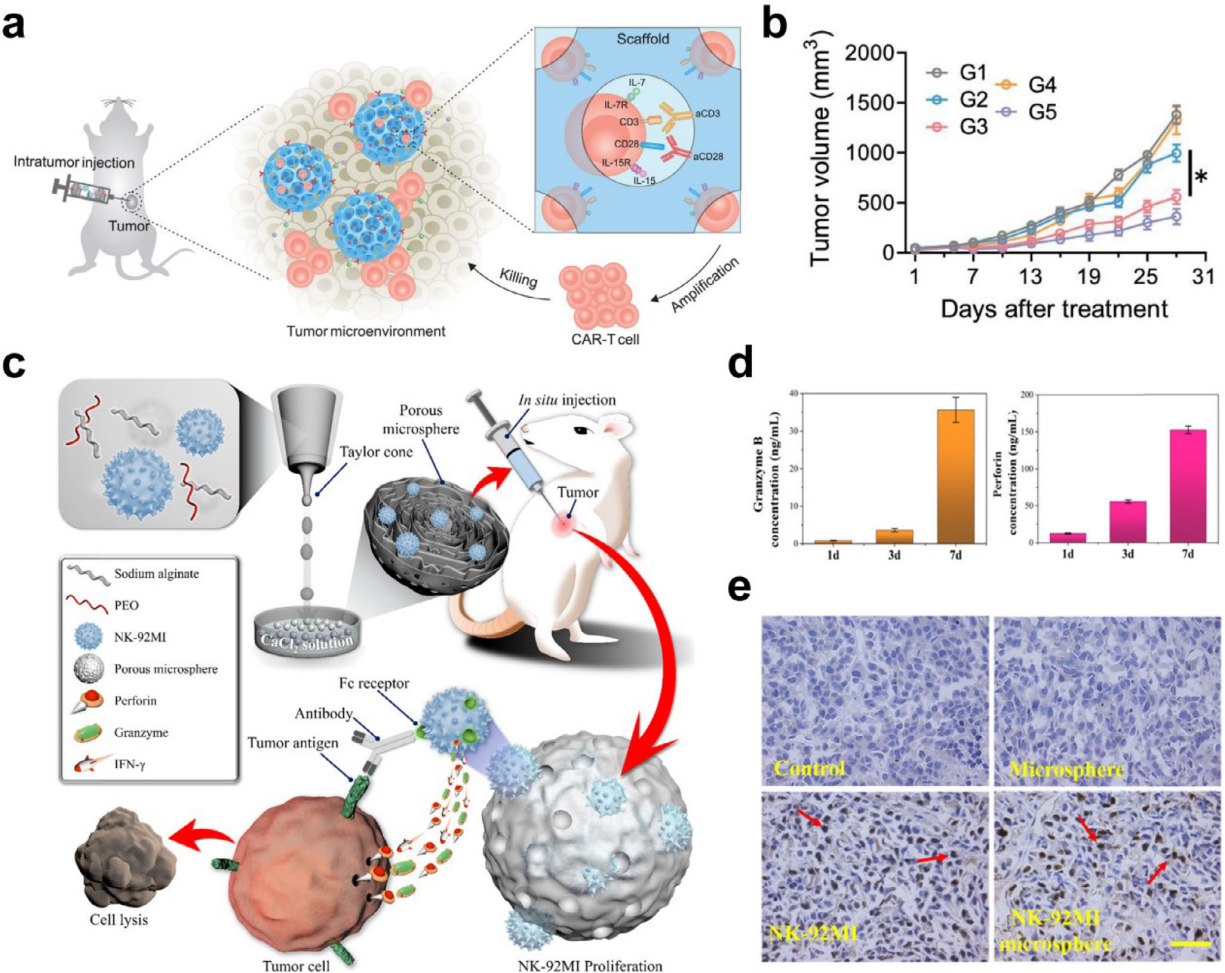
Schematic illustration of microfluidic‐derived porous microspheres for the delivery of immune cells for cancer therapy. a) Antibody functionalized porous PLGA microspheres promoted the CAR‐T cell expansion and tumor cell killing. b) Tumor growth curves (G5 represents the group of engineered CAR‐T cell‐laden PLGA porous microspheres). Reproduced with permission.^[^ ^98^ ^]^ Copyright 2024, Oxford University Press. c) In situ injection of porous Ca‐alginate microspheres loaded with NK‐92 MI cells for tumor immunotherapy. d) The level of cytotoxic factors secreted by NK‐92 microspheres. e) Apoptosis in tumor cells characterized by histological staining. Reproduced with permission.^[^ ^99^ ^]^ Copyright 2019, American Chemical Society.

The advancement of immunotherapy has paved the way for new possibilities in cancer treatment. As a personalized treatment approach, adoptive cell therapy aims to in vitro activate or modify therapeutic immune cells, including cytokine‐induced killer cells, natural killer (NK) cells, T cells, CAR‐T cells, etc.^[^ ^106^, ^107^, ^108^ ^]^ The immune cells can be reinjected into the patient to specifically target and eliminate tumor cells. Porous microspheres are excellent microcarriers for loading therapeutic immune cells. The distinctive porous structure facilitates the expansion of immune cells and allows for their interaction with tumor tissue. Furthermore, some activation signals can be incorporated onto the porous microspheres to facilitate substance transfer and proliferation of loaded cells. Taking this into account, Liao et al. employed microfluidic techniques to create poly(lactic‐co‐glycolic acid) (PLGA) porous microspheres embedded with CAR‐T cells (**Figure** **7**a).^[^ ^98^ ^]^ To foster CAR‐T cell expansion, the porous microspheres were also engineered to mimic crucial signal molecules from antigen‐presenting cells by incorporating T‐cell stimulatory signals. Notably, the expansion process endured for a month in mice with cervical cancer model and effectively inhibited tumor growth (Figure 7b). Similar to CAR‐T cells, NK cells can also eliminate cancer cells in an antigen‐independent manner. Wu et al. successfully developed Ca‐alginate porous microspheres loaded with NK‐92MI cells through microfluidic electrospray, which showed promising results in in situ treatment of breast cancer in mice (Figure 7c).^[^ ^99^ ^]^ The porous microspheres provided protection to NK‐92MI cells against the host immune rejection. NK‐92MI cells within the microspheres exhibited continuous proliferation during the culture process, accompanied by a gradual increase in the secretion of cytotoxic factors perforin and granzyme B (Figure 7d). Particularly, the NK‐92MI cells could poke out from the surface of the microspheres and make contact with surrounding tumor tissues. Apoptosis of tumor cells was the most in the NK‐92 MI microsphere group (Figure 7e).

CD8^+^ PD‐1^+^ T cells can be expanded in vitro and reinfused into body without the need for genetic modification, which has generated significant research interest among scientists. However, the expansion process often leads to increased cell differentiation, which negatively impacts anti‐tumor efficacy. To address this, Zhang and co‐workers developed a microfluidic chip that efficiently isolates and encapsulates CD8^+^ PD‐1^+^ T cells from the blood of melanoma‐bearing mice.^[^ ^109^ ^]^ The cell‐laden alginate gel microspheres, also incorporating cytokines and adjuvants, enhance immune responses and promote dendritic cells and T cells migration to the injection site. This improvement may be attributed to the confined space of the microspheres, which facilitates effective interactions between cells and adjuvants, helping to maintain lower levels of cell differentiation.

### 3.2 Tissue Regeneration and Repair

Tissues are complex 3D structures composed of various cell types and ECMs, with their functionality shaped by cell signaling and interactions with the surrounding ECMs. Common treatments for tissue damage involve surgical repair, organ transplantation, prosthetic implantation, or the use of mechanical devices to assist patients in regaining lost functions.^[^ ^110^, ^111^, ^112^ ^]^ However, these strategies present inherent limitations, such as donor shortages, immunological rejection, and difficulties with integration. Stem cells have demonstrated broad prospects for application in tissue regeneration and repair due to their self‐renewal, multi‐lineage differentiation, and immunomodulatory capacities.^[^ ^113^ ^]^ However, precisely because of the high plasticity of stem cells, there is a risk of multidirectional differentiation after transplantation, especially when the cells are not fully differentiated or when the microenvironment is not adequately controlled. To mitigate this risk, the differentiation process of stem cells must be precisely controlled. For example, specific differentiation factors, genetic engineering techniques, or biomaterials (such as microfluidic hydrogel microcarriers) can be used to ensure that stem cells differentiate in the desired direction and stably form the target cell type. Microfluidic methods facilitate the efficient organization of stem cells and biomaterials into structured scaffolds that promote the formation of microtissues capable of carrying out functions specific to the tissue type. These cell‐laden microcarriers serve as local delivery systems to prevent ectopic growth and uncontrolled proliferation of stem cells, thereby greatly reducing the risk of tumorigenesis.

For diverse tissue injuries, distinct cell delivery microcarriers offer unique advantages. Transdermal microneedle arrays loaded with stem cells are a promising device for treating chronic skin diseases like diabetic wounds. As mentioned in Section 2.3, uniform‐sized ADSC spheroid aggregates embedded in microneedle arrays were prepared for the treatment of diabetic wounds by utilizing the precise fluid manipulation of microfluidics.^[^ ^89^ ^]^ The microneedle arrays can penetrate the epidermis, enabling precise and deep delivery of ADSC spheroids. The exchange of multiple active compounds prompted the initiation of neovascularization, collagen deposition, and tissue regeneration, thereby facilitating the healing of diabetic wounds.

**Figure 8:**
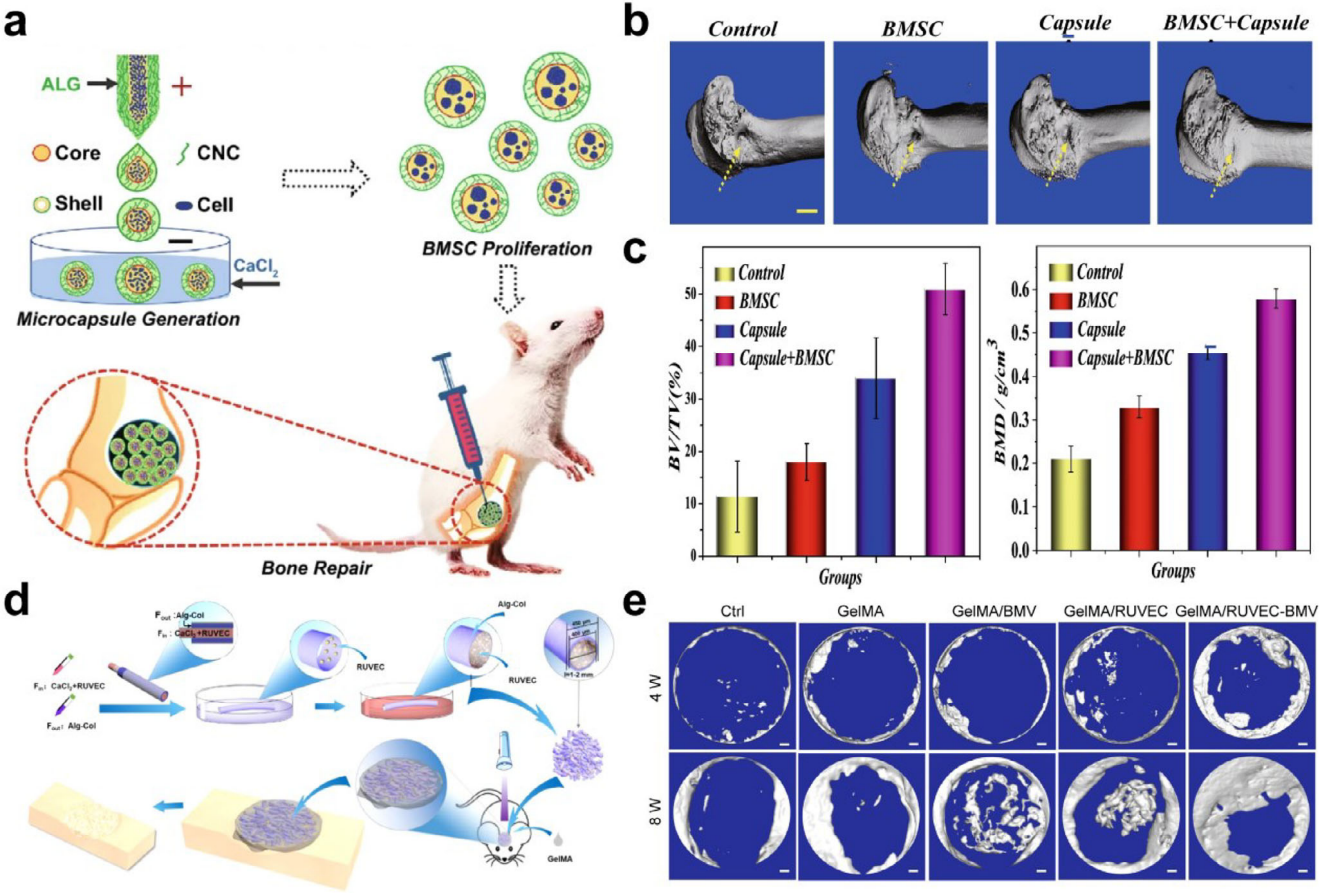
Microfluidics‐based cell‐laden microcapsules and microfibers for bone repair. a) Schematic illustration of delivering BMSC‐laden microcapsules for bone repair. b,c) Images and quantitative analysis of micro‐CT. Scale bars, 1 mm. Reproduced with permission.^[^ ^101^ ^]^ Copyright 2021, Springer Nature. d) Schematic illustration of fabrication and implantation of RUVEC‐BMVs. e) Micro‐CT images of calvarial defects with different treatments. Scale bars, 400 µm. Reproduced with permission.^[^ ^104^ ^]^ Copyright 2022, Elsevier.

BMSCs are widely used in treating bone and cartilage defects due to their ability to self‐renew and differentiate into osteoblasts and chondrocytes, directly contributing to tissue formation and repair.^[^ ^114^ ^]^ Their relative abundance and ease of acquisition through simple bone marrow aspiration make them particularly suitable for applications. For the treatment of bone defects, Yang et al. employed an all‐aqueous phase microfluidic electrospray approach to prepare core‐shell microcapsules loaded with BMSCs (**Figure** **8**a).^[^ ^101^ ^]^ Introducing cellulose nanocrystals into the shell layer roughens the surface of the microcapsules and creates small pores, which enhances the mechanical strength of the microcapsules and promotes cell growth. After injecting the microcapsules into the bone defect sites of SD rats for 8 weeks, the imaging and analysis results from micro‐CT indicated that the BMSC‐laden microcapsules effectively enhanced bone regeneration (Figure 8b), with notable improvements in bone volume fraction and bone mineral density (Figure 8c).

Facilitating neovascularization plays a pivotal role in bone tissue engineering due to the highly vascularized nature of bone.^[^ ^115^ ^]^ Microfibers loaded with endothelial cells can function as biomimetic microvessels (BMVs), expediting the process of vascularization. As a typical example, an alginate‐collagen composite hydrogel BMV loaded with rat UVECs (RUVECs) were fabricated by Wang et al. based on the microfluidic technique (Figure 8d).^[^ ^104^ ^]^ These BMVs enabled the delivery of oxygen, nutrients, growth factors, and other essential molecules to the bone tissue. Additionally, the RUVEC‐BMVs supported the formation of new blood vessels at the bone defect site. After implanted into rat calvarial defects, the micro‐CT image showed that the RUVEC‐BMVs group exhibited better bone formation (Figure 8e).

### 3.3 Gut Microbiota Restoration

The gut microbiota, a complex and dynamic ecosystem of trillions of microorganisms, plays a fundamental role in maintaining human health.^[^ ^116^ ^]^ These diverse communities inhabit the gastrointestinal tract and contribute to various physiological functions, including energy metabolism, immune modulation, and maintaining the integrity of the barrier system.^[^ ^117^ ^]^ Additionally, it helps protect against pathogens by maintaining the intestinal barrier and influencing local immune responses.^[^ ^118^ ^]^ Increasing evidence indicates that when the gut microbiota is disrupted, the risk of inflammatory bowel disease (IBD) significantly increases.^[^ ^119^ ^]^ Studies have shown that MSCs isolated from umbilical cord blood (UCB‐MSCs) offer new opportunities for treatment of IBD and restoration of healthy microbiota.^[^ ^120^ ^]^ Considering the hostile environment of varying pH, digestive enzymes, and bile salts in the gastrointestinal tract, Kim et al. developed a microcapsule containing UCB‐MSCs with an intermediate oil layer through microfluidic method to enhance the adaptability of stem cells during oral delivery.^[^ ^102^ ^]^ The thin oil layer was sufficient to isolate MSCs from the harsh gastric acid environment. Due to the relatively low stiffness of the hydrogel shell, it underwent segmental rupture along with intestinal peristalsis, allowing the delivery of UCB‐MSCs to the inflamed colon site. The results demonstrated that the released UCB‐MSCs could effectively alleviate intestinal inflammation and dysbiosis of the microbiota.

**Figure 9:**
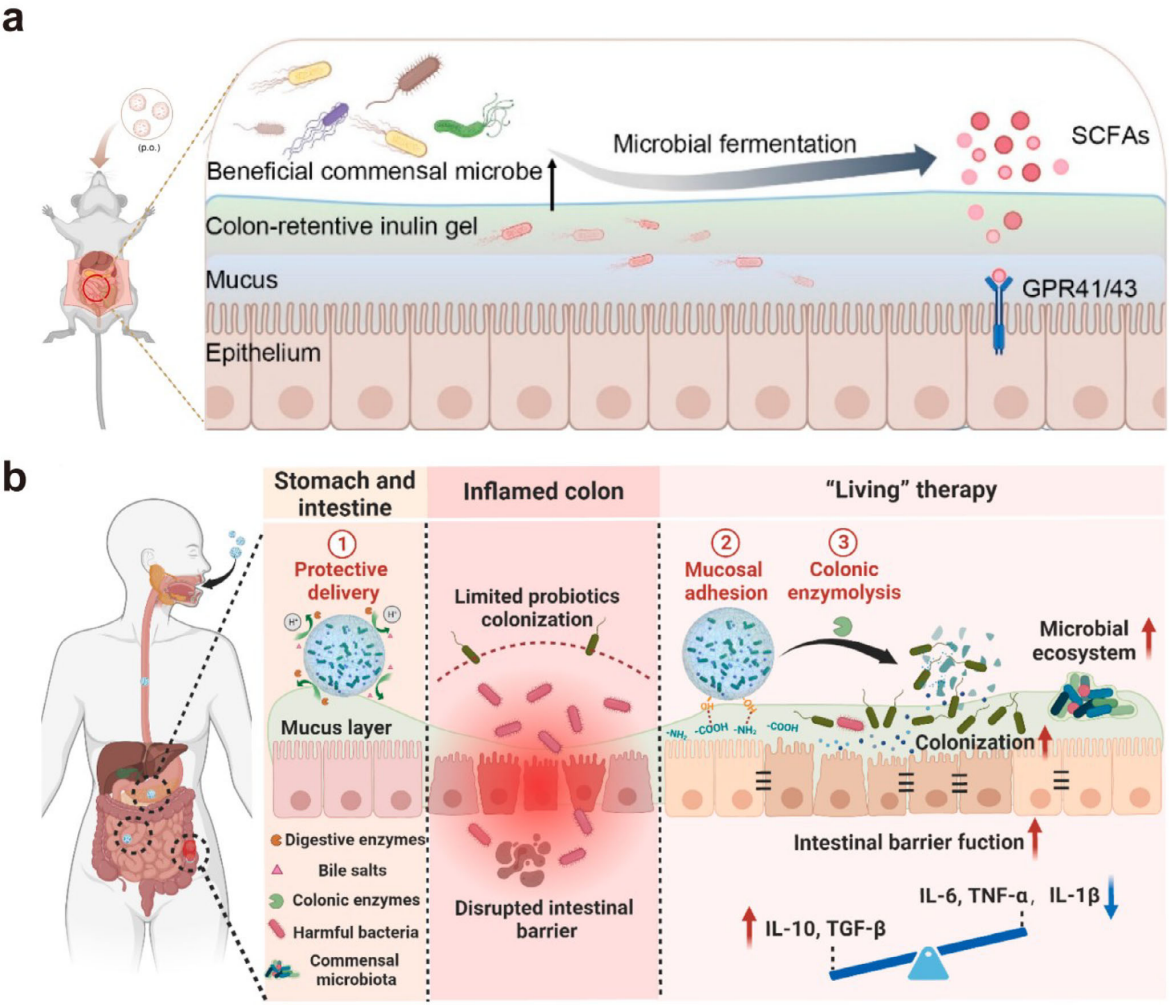
Oral delivery of probiotics‐laden microspheres for gut microbiota restoration. Reproduced with permission.^[^ ^123^ ^]^ Copyright 2024, American Chemical Society. a) Schematic illustration of colitis treatment by SCFAs produced from delivered EcN. b) Schematic illustration of colon‐targeted microspheres for gut microbiota regulation. Reproduced with permission.^[^ ^97^ ^]^ Copyright 2024, Elsevier.

In addition to stem cells, oral probiotics also has a positive effect on improving the gut microbiota.^[^ ^96^ ^]^ Probiotics themselves are unicellular organisms with the ability to grow, metabolize, and reproduce; they can also secrete bioactive factors like short‐chain fatty acids (SCFAs) and specific enzymes to inhibit the growth of harmful microorganisms.^[^ ^121^ ^]^ Probiotics have the potential to strengthen the intestinal barrier and promote optimal immune system activity.^[^ ^122^ ^]^ Based on this, Yin et al. employed Ca‐alginate and inulin to form solid microspheres, within which the probiotic *Escherichia coli* Nissle 1917 was encapsulated.^[^ ^123^ ^]^ After oral administration, the Ca‐alginate network degraded in response to the alkaline condition of intestine, and the presence of inulin enhanced the retention of EcN, as shown schematically in **Figure** **9**a. Delivery of the microspheres effectively reduced the quantity of harmful bacteria, restored the balance of the colon microbiota, and exerted therapeutic effects against IBD. In another improved study, Yang et al. developed methacrylated dextran microspheres, which served as colon‐targeted microcarriers that enabled more precisely delivery of EcN to the inflamed areas (Figure 9b).^[^ ^97^ ^]^ The integration of tannic acid component in the microcarriers enhanced colon retention through hydrogen bond interaction with the mucus layer. Triggered by colon enzymes, the composite hydrogel matrix degrades, and EcN was gradually released, thus restoring the balance of the microbiota and promoting colon barrier repair.

### 3.4 Metabolic Disorder Regulation

**Figure 10:**
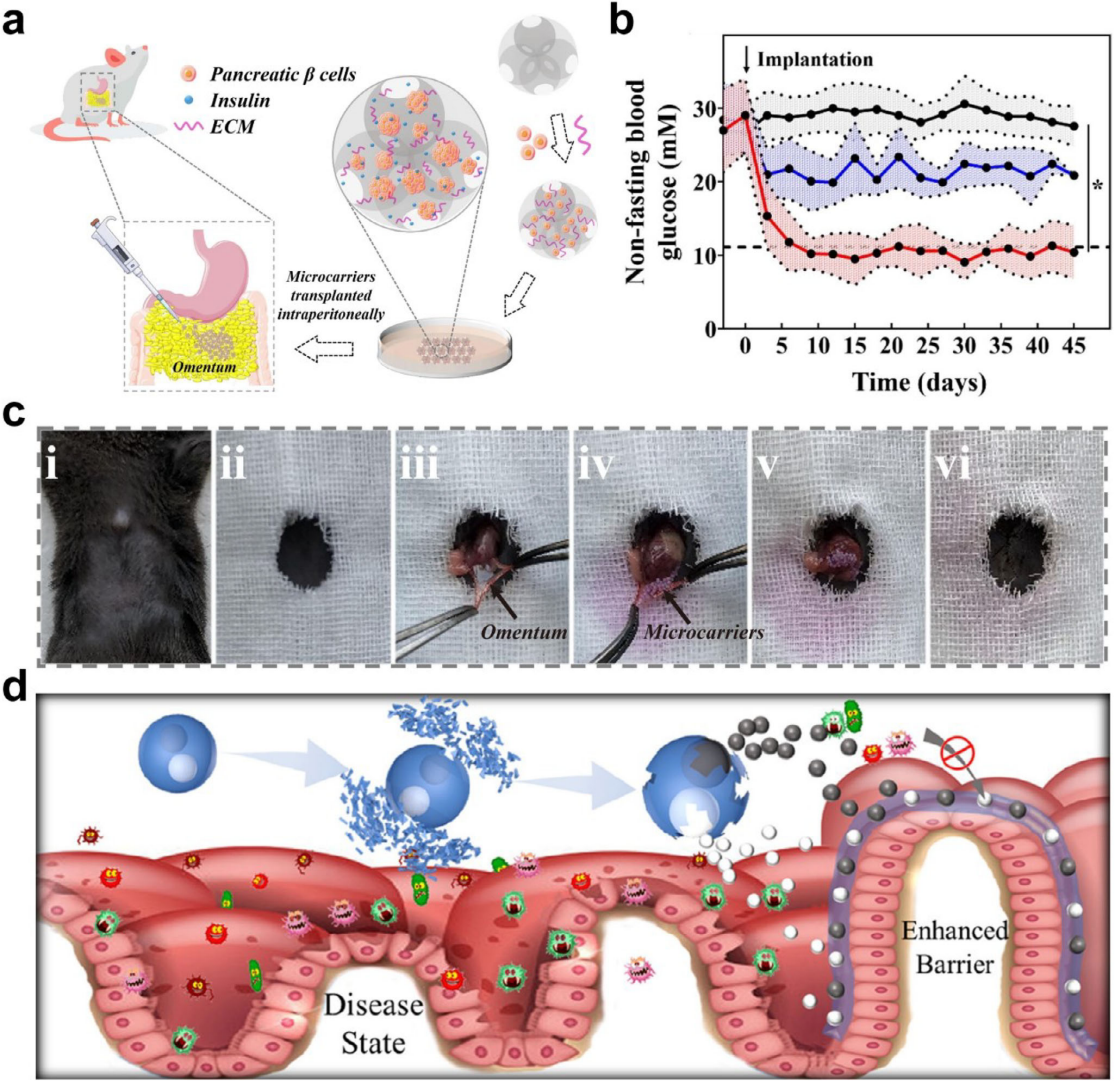
Cell‐laden microcarriers for metabolic disorder regulation. a) Schematic illustration of porous microspheres loaded with β cell aggregates for the treatment of diabetes. b) The average fasting blood glucose levels after transplantation in different groups. c) Omental transplantation of microcarriers. Reproduced with permission.^[^ ^100^ ^]^ Copyright 2022, Elsevier. d) Schematic illustration of dual‐core probiotics microcapsules for synergy therapy of metabolic syndrome. Reproduced with permission.^[^ ^103^ ^]^ Copyright 2020, Elsevier. American Chemical Society.

Metabolic disorders refer to a group of conditions that disrupt normal metabolism, the process by which the body converts food into energy.^[^ ^124^ ^]^ These disorders can affect how the body uses nutrients, leading to imbalances in essential substances such as carbohydrates, fats, and proteins. Typical diseases caused by metabolic disorders include diabetes mellitus, obesity, hyperlipidemia, thyroid dysfunction, etc.^[^ ^125^ ^]^ For diabetes treatment, current research aims to develop strategies for restoring controlled insulin secretion.^[^ ^126^ ^]^ Transplantation of islet β cells via functional microcarriers is a promising way, as β cells can sense glucose levels and intelligently release insulin, thereby reducing the risk of hypoglycemia. Li et al. prepared porous microspheres by microfluidic technology and filled the pores with β cells and ECM‐derived Matrigel (**Figure** **10**a).^[^ ^100^ ^]^ The ECM‐mimic environment preserved the initial function of β cells during cell culture and promoted the growth of β cells into 3D cell aggregates. The porous microspheres loaded with β cell aggregates were delivered to the mouse omentum (Figure 10c). Due to the protective effect of microspheres and the function of the β‐cell 3D aggregates, normal blood glucose levels were maintained in diabetic mice for at least 42 days (Figure 10b).

Microfibers have also been employed to deliver islet cells. Researchers have tried to increase the mechanical strength of microfibers to prevent transplant damage, thereby increasing the efficacy of blood glucose regulation. Ozawa et al. prepared alginate‐polyarcylamide dual‐network microfibers entrapping β cells by microfluidic method.^[^ ^105^ ^]^ The fibers can be assembled into a cylindrical hydrogel block. Transplantation of the assembled hydrogel was conducted in both subcutaneous and intraperitoneal sites of diabetic mice, revealing that the dual‐network fibers markedly reduce blood glucose levels. The dual‐network structures prevented transplant damage, as the assembled hydrogel maintained its normal morphology after being retrieved from the body. Watanabe et al. found that 1mm‐thick Ba‐alginate fibers loaded with primary islets maintained normal blood glucose levels in diabetic mice for over 100 days, which was four times longer than the duration observed with 0.35 mm‐thick fibers.^[^ ^127^ ^]^ Additionally, they provided almost the same level of glucose tolerance as healthy mice. The 1.0 mm‐thick fibers demonstrated safety as their implantation did not induce notable foreign body reactions or adherence to adjacent tissues.

In another attempt, probiotics‐laden microcapsules were utilized to treat metabolic syndrome (Figure 10d).^[^ ^103^ ^]^ Microfluidic electrospray technique was employed to simultaneously encapsulate *Lactobacillus* and *Bacillus subtilis* in separate compartments within dual‐core microcapsules. Contact inhibition of the two probiotics was effectively prevented due to physical isolation. At the same time, the *Bacillus subtilis* could consume oxygen, which provided a favorable environment for the anaerobic *Lactobacillus*. After delivery of the microcapsules, both hepatic fat deposition and intestinal barrier damage in mice with metabolic syndrome induced by a high‐fat diet were significantly ameliorated.

As mentioned above, the structural type, size, porosity, and surface characteristics of the microcarrier can be freely tuned using microfluidics to achieve the best match with live cells, thus providing the most suitable delivery system for a varieties of disease conditions. Bioactive molecules and growth factors can be easily incorporated to the microcarriers to perform a synergistic effect. Another key benefit over traditional cell delivery methods is the consistency of each batch of microcarriers, which is a decisive factor in achieving consistent and reliable treatment outcomes with each dosing. The precise controllability of microfluidics could be the greatest advantages for the derived cell‐laden microcarriers in terms of future clinical translation.

## 4 Conclusion and Perspectives

In conclusion, microfluidics‐based cell‐laden microcarriers, including microspheres, microcapsules, microfibers, and microneedles, represent a groundbreaking approach for live‐ cell delivery, providing unparalleled precision in fabrication and enhanced control over cellular microenvironments. Microcarriers prepared by microfluidics exhibit uniformity in size with specific geometries and surface properties, which are crucial for optimizing cell adhesion, proliferation, and functionality. Each microcarrier serves as an identical but independent functional unit, which enables the co‐encapsulation of live cells with growth factors, antibodies, immunoadjuvants, or other bioactive substances to enhance therapeutic efficacy. The ability to manipulate flow rates and channel dimensions enables the engineering of microcarriers tailored for various cell types, ensuring compatibility with diverse therapeutic applications.

Although great progress has been achieved in microfluidics‐based microcarriers for delivering live cells, challenges remain for practical applications. First, the functions of tissues and organs depend highly on intercellular communication and cell‐ECM interactions. While traditional biomaterials may possess mechanical properties similar to ECM, they are insufficient in replicating the intricate structural and compositional features of native tissues and organs.^[^ ^128^ ^]^ Studies suggested that integrating ECM components into microcarriers or chemically modifying carrier materials can improve cell survival rates.^[^ ^37^, ^38^, ^83^ ^]^ In the future, leveraging microfluidic technology to construct more intricate microcarrier structures and accurately regulate the spatial arrangement of different cell types within microcarriers may enhance cell growth and reconstitute tissue‐level functions. Second, microfluidic encapsulation of cells in microspheres or microcapsules involves random entry of cells into droplet templates, with cell number in each droplet following a Poisson distribution.^[^ ^129^ ^]^ This inherent randomness poses challenges in precisely controlling cell numbers within droplets. Thirdly, microcarriers are typically generated in an oil continuous phase and require post‐processing steps like washing, transferring to an aqueous phase, and centrifugation for subsequent use. However, complete removal of the oil phase can be challenging, and these post‐processing steps may impact cell viability. In view of this, more efforts can be devoted to developing microcarriers derived from all‐aqueous phase emulsions.

Typically, by functionalizing the surface of microcarriers, their adhesion, stability, and targeting performances can be improved, thereby optimizing the behavior of preparations in vivo. In addition to physical encapsulation, alternative modes of interaction between microcarriers and live cells, such as chemical conjugation and electrostatic adsorption, are expected to achieve smart release of cells in response to fluctuations in the microenvironment, including pH, redox status, enzyme activity, and hypoxia). Additionally, exploring the synergy between microfluidics and 3D bioprinting could lead to the creation of layered materials and cells in complex configurations that support intricate tissue architectures, thereby broadening the applications in regenerative medicine. This integration enhances the design of cellular microcarriers by enabling the incorporation of multiple cell types and the precise placement of biochemical cues. Moreover, considering the expanding scope of cell therapy, some inactivated cells can be encapsulated within microfluidic‐derived microcarriers to serve as tumor vaccines, which can recruit and activate immune cells for tumor immunotherapy. Dormant probiotic spores can also be delivered into the body and germinate in appropriate environments to exert therapeutic effects.

Attention should also be given to the scalability of microfluidic systems. While these technologies excel in small‐scale applications, transitioning to larger volumes for clinical or industrial use poses challenges in maintaining reproducibility. Innovations such as modular microfluidic designs could offer solutions, enabling parallel processing without compromising quality. 3D printing technology can help create microfluidic channels with consistent quality, thereby addressing issues of device clogging and replacement due to excessive residues after multiple uses. Refining production methods to ensure consistent quality and reproducibility will facilitate regulatory approval and clinical translation. Emphasizing user‐friendly systems for easy implementation in clinical settings can bridge the gap between innovative research and practical application, ultimately unlocking the full potential of this technology in regenerative medicine and beyond. Overall, microfluidics‐based microcarriers offer a powerful approach for cell delivery and disease treatment. With advancements in cell biology, materials science, and microfluidics, it is believed that this field has broader prospects and applications.

## Conflict of Interest

The authors declare no conflict of interest.
